# Can Voluntary Health Insurance for Non-reimbursed Expensive New Treatments Be Just?

**DOI:** 10.1093/phe/phad015

**Published:** 2023-07-16

**Authors:** Jilles Smids, Eline M Bunnik

**Affiliations:** Department of Medical Ethics, Philosophy and History of Medicine, Erasmus MC, University Medical Centre Rotterdam, Rotterdam, The Netherlands; Department of Medical Ethics, Philosophy and History of Medicine, Erasmus MC, University Medical Centre Rotterdam, Rotterdam, The Netherlands

## Abstract

Public healthcare systems are increasingly refusing (temporarily) to reimburse newly approved medical treatments of insufficient or uncertain cost-effectiveness. As both patient demand for these treatments and their list prices increase, a market might arise for voluntary additional health insurance (VHI) that covers effective but (very) expensive medical treatments. In this paper, we evaluate such potential future practices of VHI in public healthcare systems from a justice perspective. We find that direct (telic) egalitarian objections to unequal access to expensive treatments based on different ability to afford VHI do not stand up to scrutiny. However, such unequal access might lead to loss of self-respect among individuals, or loss of fraternity within society, rendering it more difficult for citizens to interact on equal moral footing. This would be problematic from a relational egalitarian perspective. Moreover, the introduction of VHI might turn out to have negative consequences for the comprehensiveness and/or the quality of the public healthcare services that are offered to all patients equally through basic health insurance. These consequences must be weighed against potential health gains and the value of liberty. We conclude that governments should be careful when considering the introduction of VHI in public healthcare systems.

## Introduction

More and more medical treatments are entering the market that are effective, yet so expensive that they are insufficiently cost-effective to be covered by publicly funded healthcare. For example, the Dutch government recently decided not to fund Trodelvy for patients with triple-negative breast cancer. Based on the list price, Trodelvy would cost EUR 69.000 for 5,4-month overall survival, and price negotiation did not result in a (confidential) agreement.^1^ Immunotherapies in cancer care, such as Yescarta, which is potentially curative, may cost around €400.000 for one patient, including the costs of additional care associated with the treatment (Hernandez *et al.*, 2018).^2^ The costs of these immunotherapies may easily be considered as too high by priority-setting agencies in publicly funded healthcare systems. Another class of effective but very expensive treatments are gene therapies. Over the next few years, many new innovative medical treatments are expected to be approved for marketing which will put healthcare budgets under increasing pressure.

If these treatments are insufficiently cost-effective, it is perfectly just and fully rational not to include them in publicly funded healthcare made available to all. Societal resources are inevitably limited and governments have many other responsibilities, such as providing (funding for) education, safety, housing, etc., which are no less important. Moreover, even if health were treated as the most important value, it is well-known that good education and housing are among the social determinants of health. So, even if health were top priority, it would be unjust and irrational to transfer unlimited amounts of funding from education and housing to healthcare so as to include ever more expensive treatments in publicly funded healthcare.

Now, when effective but too expensive treatments are not reimbursed, or not yet reimbursed, pending price negotiations, individual patients may still want access to these treatments. In such situations, they may seek access by paying out-of-pocket. Since only a very small minority has the means to do so, a demand will likely emerge for voluntary health insurance (VHI) that covers such treatments. Given certain favorable societal and market conditions, insurers will respond by offering this insurance to consumers. In the UK, this has indeed happened in response to a policy that was introduced in 2008 and allowed for out-of-pocket payment for expensive non-funded cancer treatments. In the UK, VHI for such cancer treatments seems a socially accepted practice.^3^

However, the introduction of VHI will lead to unequal access to treatments among patients equally in need, based on their different ability to afford the monthly premiums for VHI. And this raises our paper’s question: how should we evaluate, from a justice perspective, practices in which some share of citizens can take out VHI while others lack the means to do so? Should citizens be granted the liberty to spend their after-tax income on buying additional health insurance that covers expensive treatments, or would that lead to objectionable inequality?^4^ These are the questions we address in this paper.

In the section *When Would There Be a Demand for VHI, and How Would It Work?* we explain in more detail when and why insurers would start to offer VHI for expensive treatments that are non-reimbursed. In the section *In-Principle Justice-Based Considerations with Respect to Allowing VHI*, we evaluate such VHI from the perspective of equality and liberty and ask whether there are what we will call ‘in-principle justice-based objections’. In the section *Does VHI Lead to Relational Inequality?* we discuss whether in practice a society in which a minority of citizens cannot afford VHI will suffer from social inequalities of the sort that relational egalitarians would object to. In the section *Is VHI for Expensive Treatments Detrimental to Egalitarian Healthcare Systems?* we briefly discuss whether the introduction of VHI would give rise to changes in the operation and quality of the publicly funded healthcare system that would unjustly disadvantage those who cannot afford VHI. In our *Concluding Discussion*, we weigh all considerations and attempt to answer the question as to whether and when VHI can be just.

In our paper, we focus on egalitarian publicly funded healthcare systems, such as those in the Scandinavian countries, the UK and the Netherlands. These healthcare systems commonly offer, as matter of justice, a comprehensive basic package of cost-effective medically necessary healthcare to all patients for free at the point of delivery. While sometimes things like dental care are not included in this basic package, it is not customary in these systems for patients to pay for expensive medically necessary treatments out-of-pocket. These systems are characterized, thus far, by the absence of a substantial private healthcare sector. These systems can be single-payer and tax-funded, funded through (mandatory) social health insurance, or some mixture of these. Therefore, whenever we speak of non-reimbursed treatments, we mean to include non-funded treatments. Throughout our paper, we will assume a sufficiently just society, in which the distribution of income levels, and the ways in which wealth is acquired, are by and large just. In our concluding discussion, we relax that assumption and explain how it alters our answer to the paper’s questions.

## When Would There Be a Demand for VHI, and How Would It Work?

A demand for VHI would emerge when countries’ priority-setting agencies would decide, on a regular basis, not to reimburse effective but very expensive treatments. We understand effective but very expensive treatments to be treatments that provide more than marginal benefit, e.g. they significantly prolong life, improve quality of life or even cure patients, but which are insufficiently cost-effective.

Priority-setting agencies in the publicly funded healthcare systems mostly have very similar criteria for priority setting: the severity of the disease or condition, the effectiveness of the treatment and its cost-effectiveness (e.g. Ministerie van Volksgezondheid, 2017; Rumbold *et al.*, 2017). These criteria are applied in conjunction: a treatment should not only be effective, but also have an acceptable cost, i.e. be sufficiently cost-effective. While the employment of cost-effectiveness analysis is widespread, most countries do not employ explicit willingness to pay thresholds per quality-adjusted life year (QALY) gained, but, rather, rely on implicit thresholds (Luyten and Denier, 2019).

Priority-setting processes generally involve further criteria or ‘social values’ that may be taken to justify higher cost per QALY. Examples include higher willingness to pay for orphan drugs (in the Netherlands), or for drugs that serve to reduce health inequalities (in the UK, see Rumboldt, op. cit., table 3). Nevertheless, these further criteria are not meant to give priority-setting agencies leeway in justifying provision of treatments associated with extremely high costs. When priority-setting processes proceed according to their design, from time to time, effective treatments *will* receive negative judgments because of insufficient cost-effectiveness.^5^

If governments start to exclude such effective treatments from the basic package, to contain spending of collective funding on healthcare, then a demand for VHI likely will emerge. We do not predict that this will happen very soon, or in all countries, for governments will search for ways to avoid decisions not to reimburse treatments, which are often unpopular. Yet, governments have sound moral reasons to stick to their priority-setting procedures and once they do, a growing number of effective treatments for severe diseases will no longer be available in the basic package. Then, because some share of the citizens will want access to non-reimbursed treatments, but lack the means to pay for them out-of-pocket, probably a demand for VHI will emerge to which insurers will respond. In fact, as we noted above, in the UK some insurers already provide VHI for non-funded cancer treatments.

The precise form of the VHI-products insurers will offer depends on a number of considerations. VHI qualifies as complementary insurance, because it covers treatments that are not included in the publicly financed basic package (cf. Sagan and Thomson, 2016: 10). In the EU, complementary health insurance is treated as a commercial product, for which governmental regulation in principle is prohibited (though in practice, some regulation seems permissible) (Sagan and Thomson, 2016: 87). This means that insurers are free to accept or refuse individual applications for insurance, to rate the premium according to individual health risk (dependent on age, sex and health condition), to exclude pre-existing conditions or charge higher premiums, and to set age limits, often 65 years. These freedoms are essential for insurers to survive in a commercial market, for they are indispensable to avoid or mitigate adverse selection. This regards the phenomenon that individuals with a higher risk of ill-health are relatively more prone to take out insurance, which leads to more spending and thus to higher premiums, which renders insurance less attractive to those at lower risk and so on. Group contracting (i.e. collective insurance) is a way to deal with adverse selection, and, moreover, to spread the risk, for instance via the employer, who may provide financial incentives to employees to join.

In a system in which insurers offer VHI that covers very expensive non-reimbursed treatments, whether individual or group insurance schemes or both, not all citizens will take out VHI. The unemployed and the retired cannot join collective schemes offered by employers. And individually, those with low incomes may not be able to afford the premiums for VHI, especially not if they are older or have pre-existing health risks that drive up their premiums. For the employed with low incomes, a good case can be made that taking out VHI is not in their best interest. It may put them under financial strain, which can be stressful, and moreover, in order to pay for the premiums, they may have to cut other expenses that might contribute (more) to a worthwhile—and healthy—life. Thus, for some, VHI will not be accessible. To what extent would that raise problems of justice? That is the question to which we will now turn.

## In-Principle Justice-Based Considerations with Respect to Allowing VHI

Let us assume that in a country with a publicly funded healthcare system, it would indeed regularly happen that effective but too expensive treatments are not included in the basic package of healthcare available to all citizens. Would it then be unjust in principle if some citizens would secure access to these treatments by taking out VHI, which other citizens cannot afford? What we mean by an in-principle justice-based consideration is a consideration that applies even if (unrealistically) nothing would change in the structure, operation and quality of the current healthcare system. We discuss two such considerations: first, unequal access to medically necessary medical treatment as objectionable from an egalitarian point of view, and second, the liberty to spend one’s means on goals as one sees fit, including healthcare.

We start with analyzing the problem of unequal access from the perspective of egalitarianism, more specifically, intrinsic egalitarianism. Intrinsic egalitarianism, or what Parfit calls ‘telic’ egalitarianism, is the view that equality is intrinsically valuable (Parfit, 1997; Temkin, 2003). On this view, if some citizens receive treatment covered by VHI, but other citizens do not receive the same treatment because they cannot afford VHI, this inequality is bad. That does not immediately imply that telic egalitarians must oppose a system in which VHI has a place. Telic egalitarians generally accept values other than equality, such as health. For, if only equality would count as a value, telic egalitarians could not hold that a situation in which two people are equally healthy is better than one in which two people are equally unhealthy. Therefore, any sensible intrinsic or telic egalitarianism is a pluralist view and, consequently, will agree that the health gains available to those with VHI are valuable.

However, it is even questionable whether equality indeed has intrinsic value. This is the point of the well-known leveling down objection, which targets the core idea of telic egalitarianism. This objection holds that if we ‘level down’ the group that is better off in order to reach a state in which all are equally well or perhaps badly off, we have worsened the situation of some while we have made no one better off. For without access to treatment, no one will get well. Hence, even though we have achieved equality, we have done nothing good, and therefore, so the leveling down objection goes, equality has no intrinsic value (cf. Parfit, 1997). This is a problem for telic egalitarians, because they must hold that leveling down is better *in one respect*. Egalitarians reply that equality does have intrinsic, *impersonal* value, and that the situation after leveling down is still better in that respect: it is more equal (Eyal, 2013). Interestingly, many physicians in a country with an egalitarian publicly funded healthcare system seem to support leveling down in order to prevent situations in which ability to pay determines which patients do and which patients do not have access to medical treatments. These physicians cited equality and equal access to healthcare regardless of ability to pay as their fundamental commitments (Bomhof *et al.*, 2022). So, intuitions on the intrinsic value of equality differ.

Therefore, we will now give two arguments that are compatible with the view that equality has intrinsic value. Both support the view that telic egalitarianism does not imply a rejection of a healthcare system that accepts and integrates VHI for expensive treatments. First, if inequality is intrinsically bad, we should take into account all relevant inequalities. Now, severely ill persons are significantly worse off than healthy citizens. In case the ill person received treatment covered by her VHI, and she were cured completely, her level of health would become equal to that of her healthy fellow-citizens.^6^ At the same time, inequality would have arisen between her and those among her ill fellow-citizens who could not afford VHI. Her health status would have become less equal to that of her ill fellow-citizens but more equal to that of her healthy fellow-citizens. Our evaluation of these changes in equality across the entire population depends on our intuitions and how we measure (in)equality. For example, if we were to employ the Gini coefficient to measure inequality (cf. Regidor, 2004), we would conclude that VHI-covered treatments that cure some very ill patients even *reduce* inequality. Hence, from the perspective of telic egalitarianism and equality as an impersonal value, there are no straightforward objections to VHI for expensive treatments that are not included in the basic package. Telic egalitarianism may even favor VIH.

Second, as already noted, any plausible telic egalitarianism is a pluralist view, which will also value improvements of the well-being of people. And in the majority of cases, health improvements may outweigh the value of equality. Many treatments, unfortunately, benefit only a subgroup of patients, or benefit patients differentially. Yet, this is no reason to withhold treatment to all (Eyal, 2013). Now, it is very hard to say in abstract terms how much weight equality should have compared to health gains. It seems to us that the practical reasoning in which this balance must be sought is very much influenced by the context. In this case, the context is a publicly funded healthcare system in which there is already, as a matter of justice, a rather comprehensive basic package that includes most (or all) cost-effective essential healthcare. This means that a lot has already been done to ensure equal access to good healthcare for all citizens. In this context, some measure of inequality due to differential access to VHI need not imply substantial overall inequality in access to healthcare: citizens who do not have or cannot afford VHI will still have equal access to good healthcare. In sum, the objection on the basis of intrinsic egalitarianism does not stand up to scrutiny: it is by no means clear that VHI would increase overall inequality, and insofar as it would, it is plausible to claim that this increase is outweighed by the health gains that result from VHI-covered treatments.

We will be brief on liberty as a consideration in favor of the availability of VHI. The countries this paper focuses on are liberal democracies in which it is deemed important to have the liberty to spend one’s after-tax income and one’s wealth on purposes of one’s choice. Accordingly, denying citizens the freedom to spend their money on VHI for expensive treatments requires substantial justification. It will not suffice to just claim that health is a special good, and therefore, access to health should not depend on ability to pay. One reason is that the members of society already have made significant contributions, by way of taxes and/or premiums to uphold a good quality healthcare system available to all. As said, another reason is that healthcare is not the only so-called determinant of health: education, housing and social class are all strongly dependent on income and wealth and all determine good health. So, it requires further justification to single out healthcare as special and ‘not for sale’.

In sum, liberty provides a strong argument in favor of VHI for non-reimbursed expensive treatments, whereas valid objections cannot be derived from telic egalitarianism. Yet, in practice, the social inequality that might result from VHI might still be experienced as problematic and regarded as unjust. This is the topic of the next section.

## Does VHI Lead to Relational Inequality?

Even though the in-principle objections to VHI on the basis of telic egalitarianism were found unconvincing, in practice, unequal access due to VHI may still be judged problematic. This might be explained with the help of relational egalitarianism. In contrast to versions of egalitarianism that focus on the equal distribution of some good (e.g. resources, opportunities, etc.), relational egalitarianism does not focus on equality as such, but on egalitarian social relations between citizens.

Authors discussing what egalitarian relations look like, generally characterize these relations in terms of what they are not: egalitarian relations are not compatible with *inter alia* certain status differences, domination, servility, social stigma, and lack of self-confidence and self-respect (Anderson, 1999; e.g. Wolff, 2015). For example, in class societies, status differences directly violate relational equality, since citizens cannot relate to each other on a basis of equal standing. Moreover, the inherently hierarchical relations in class societies involve domination, that is, the arbitrary power of higher-placed citizens to interfere with the free agency of lower-status citizens. This need not necessarily, but easily does, lead to servility, which violates relational equality and its underlying core value of equal moral worth. In such societies, it is also difficult for those who are lower ranked to maintain a healthy sense of self-respect and self-confidence, which are needed to carry out their life plans in cooperation with fellow-citizens on a basis of equal footing (cf. Rawls, 1971). And finally, those who are ranked lower may experience public stigma, which also undermines and violates social equality.

A first step in assessing the impact of VHI on social equality is clarifying what society ought to provide in order to avoid the various manifestations of relational inequality just introduced. In this respect it is important to note that unlike most other egalitarian views, relational egalitarianism is not in the business of defining something, e.g. opportunities, welfare or resources, that should be distributed in egalitarian ways. So, it is not a distributional view of justice. Instead, it is the view that what citizens owe to each other is to ensure that they can live together on a footing of equality (Anderson, 1999). This requires securing good education to develop one’s capacities, decent jobs that are part of the cooperative scheme of society, unconditional access to adequate healthcare and more.

Once society has fulfilled its duty to all citizens to provide those goods, including comprehensive healthcare, if relational inequality were to emerge as a result of allowing insurers to offer VHI, which not all citizens can afford, it is not clear whether this would mean that society fails those citizens (cf. Voigt and Wester, 2015: 212). To answer this question, more analysis is needed, both empirical and normative.

The most important empirical questions are whether and to what extent those citizens will suffer a loss of self-respect, and whether unequal access to VHI will lead to an unacceptable ‘loss of fraternity’ (Scanlon, 1996: 11) within society. In her discussion of two-tiered healthcare systems, Fourie suggests that the more substantial the benefits that are available only on the second tier, the more plausible it becomes to think that ‘people feel inferior’ and ‘civic friendship’ is interfered with. For, they are no longer ‘in the same boat’ (Fourie, 2016: 200). People’s experiences of loss of self-respect or loss of fraternity will likely be affected by the share of citizens that cannot afford VHI. In case, say, only 20 per cent of the citizens could afford VHI, then the very large majority of citizens would still be in the same boat. This would be different if, say, 80 per cent of citizens VHI could afford VHI, but 20 per cent could not.^7^ Other factors concern whether children are involved or mainly elderly patients, how great the medical benefit of treatment is, how visible differential access to treatments will be and on what scale it will happen.

The normative questions are, firstly, whether the individual and social evaluations involved in experiences of loss of self-respect and of fraternity, respectively, are justified or, rather, constitute an ‘evaluative error’ (Scanlon, 2018: Ch. 3), and secondly, how loss of self-respect and of fraternity, insofar as they indeed happen to occur, should be weighed against the health gains and the value of liberty?

To illustrate, think of Zolgensma (Onasemnogene abeparvovec), an innovative gene therapy that halts the progression of, and possibly even cures, spinal muscular atrophy (SMA). For children with this rare genetic condition, timely treatment with Zolgensma means, hopefully, that they will have a relatively normal development rather than never being able to walk and eventually needing ventilation. Zolgensma, however, has a very high listed price of 1,94 million euro.^8^ In the Netherlands, Zolgensma was not available for more than a year until the government and the pharmaceutical company reached an undisclosed price agreement.^9^

Now, suppose that during that time, some parents of children with SMA had taken out VHI that included their children and covered Zolgensma, so their children could receive timely treatment, while other parents did not have VHI and their children could not be treated. How would such a situation be experienced by the parents without VHI, their direct social environment and society at large? In any case, the stories of parents of children with SMA who tried to raise 1,94 million euro by means of crowdfunding when Zolgensma was not yet reimbursed, clearly testify to their distress.^10^ We speculate that in situations like those in which children with SMA would not have access to Zolgensma because of lack of VHI, parents may suffer, also from self-blame or negative self-appreciation and loss of self-respect.

We also think that in egalitarian societies it is likely that fellow-citizens will find it very hard to see that VHI status would make such great difference to the health and lives of children with diseases like SMA. There could easily emerge a sense that decent societies should not allow such differences. Citizens might come to see their society as insufficiently caring for its children. In these ways, their understanding and self-image of living in a society of free and equal citizens (fraternity) may come under strain. Here the well-known mechanisms described by Jonsen under the heading of the ‘rule of rescue’ likely are at work: faced with fellow humans who face illness leading (in due course) to death, we find it psychologically very difficult to do nothing (Jonsen, 1986).

This effect may be stronger in some cases than in others: for instance, it may turn out that VHI that causes differential access to gene therapies such as Zolgensma for children will not be socially accepted, while private insurance such as in the UK for cancer treatments that are not available on the National Health Service (NHS) *is* socially accepted. There are some indications that the latter might indeed be the case.^11^ However, this might merely reflect the fact that the large majority of effective cancer treatments are still available, either on the NHS, or via the alternative routes such as Individual Funding Requests.^12^ For these cancer patients, there are alternative treatment options available, and the new treatment may not be as effective (as is Zolgensma for SMA patients). Societal acceptance might very well change under the circumstances that more effective treatments will be judged simply too expensive to offer on the NHS. To summarize, a practice of differential access to VHI in egalitarian societies may harm relational equality by giving rise to loss of self-respect and loss of fraternity resulting from a public sense of caring too little for fellow-citizens.

Turning to the first of our two normative questions, would these responses not be based on an evaluative error? If one is simply doing one’s job and earns a living for one’s family, there is no reason to blame oneself for not earning enough to be able to afford VHI. Here the myth of meritocracy is relevant: it is a mistake to blame it on a person’s supposed lack of effort that he does not earn a larger income. Similarly, the sense of caring too little involves an evaluative error, because citizens *do* care by collectively funding healthcare for all.

One might object that *feelings* of loss of self-respect or fraternity are not the real issue. Perhaps these feelings are merely psychological responses to an underlying problem, namely: that differences in access to medical treatments, caused by the introduction of VHI, are unjust or unfair? Above, we have argued that VHI is not unjust, neither on a societal level nor on an inter-individual level. Note again that society has already gone to great lengths to provide good quality and comprehensive healthcare to all of its members. In this way, citizens express their concern to live with one another as free and equal fellow-citizens. Given limited resources, governments must rightfully limit their healthcare expenses. Therefore, it is not unfair when not all effective treatments are publicly funded. The need to limit healthcare expenses follows from all theories of justice. None of these theories provide a valid justice-based argument against allowing VHI.

Can it be considered unjust when *individuals* take out VHI for non-funded treatments? Assuming just income distributions, it is hard to see that individuals who take out VHI would be treating their fellow-citizens unfairly, as they have already done what they owe to their fellow-citizens, i.e. contribute to the publicly funded healthcare system. As we argued above, curtailing these citizens’ liberty to spend their resources to secure access to expensive treatments requires justification, which is difficult to provide based on existing theories of justice.

Yet, realizing all this may not suffice to lessen the perception that less affluent patients, or children of less affluent citizens, are abandoned by their fellow-citizens. Underlying this may be the feeling that their fundamental moral equality has been violated.

Therefore, turning to our second normative question, when the harms to social equality are non-negligible, how should they be weighed? The fundamental question here is how to weigh real losses in relational equality that are, however, not reasonable in the sense that they originate at least partly from evaluative errors? On the one hand, limiting healthcare expenses is necessary in order to provide other goods essential to relating to one another as equals, but on the other hand, real losses of self-respect and fraternity simply undermine relational equality. In any case, it will be very hard to directly address and correct the evaluative errors involved. Moreover, the solidaristic motives underlying the sense of fraternity of citizens that would oppose VHI are valuable in themselves.

We leave this fundamental question for another occasion and note that, from a more pragmatic point of view, governments seeking support for their policies simply have to deal with these societal sentiments. The negative impact on relational equality, then, must be considered in the moral evaluation of VHI. That is, relational inequality should be taken into account together with beneficence and liberty, and potential negative consequences of VHI for the publicly funded healthcare system. To these latter considerations, we will now turn.

## Is VHI for Expensive Treatments Detrimental to Egalitarian Healthcare Systems?

In this section, we will look at three other in-practice justice-based considerations that might count against VHI for expensive non-reimbursed treatments. These considerations arise from how VHI might have negative consequences for publicly funded healthcare systems. First, it might affect the coverage of mandatory basic health insurance. Second, treatments reimbursed under VHI could lead to displacement of healthcare that is part of the basic package. Third, having to treat patients in equal need differently might be too hard for healthcare workers, because it goes against their egalitarian ethos.

The availability of VHI as an alternative way to access expensive treatments may lessen the pressure on governments to maintain a comprehensive basic package (cf. Fenton, 2011). Higher-income citizens who are best positioned to exert influence will be able to take out VHI, which probably will weaken protest from their side, which might result in a poorer basic package. Alternatively, the opposite effect might happen if the apparent effectiveness of a VHI-covered treatment leads to pressure on governments to include it in the basic package available to all citizens. Yet governments will probably lose bargaining power in price negotiations with pharmaceutical companies if these can also do business with insurers that offer VHI (Bloor, 2008). How these scenarios will interact and work out together is yet another empirical question.

To complicate matters, a somewhat less comprehensive basic package is not evidently bad. For citizens of the lower income groups, who are the primary concern of egalitarians, this might in fact be preferable. For these groups it might actually be good if some expensive treatments were only available on VHI. This is because the balance that governments currently strike between expenses for healthcare and other social goods, especially education and housing, may not be optimal for them. That is, as a group, they might in fact be better off, and their lives and those of their children might go better, with better education and housing, even if at the cost of somewhat less comprehensive healthcare. Alternatively, they can be compensated for lack of access to some treatments by decreased cost-sharing, e.g. through lowering of mandatory deductibles, which would benefit them in terms of increased discretionary income.

However, to prevent the emergence of an ethically problematic two-tiered system, the basic insurance package should remain sufficiently comprehensive and of good enough quality to remain attractive to all citizens (Krohmal and Emanuel, 2007). If it does not, wealthy citizens have an incentive to withdraw their support for the first tier and prefer a completely private parallel healthcare system. If a substantial private tier were to emerge, and the financial solidarity of the wealthy with those of lower socio-economic status were to dissipate, the quality and comprehensiveness of publicly funded healthcare would suffer severe blows. Whether this will happen is in the hands of governments and healthcare authorities. Remember that we started with the assumption that VHI covers treatments that are effective yet simply too expensive to be included in the basic package. Therefore, as long as current priority-setting procedures and criteria remain in place, the current basic package remains attractive and VHI merely offers additional, insufficiently cost-effective medical treatments. In sum, the introduction of VHI in egalitarian healthcare systems need not threaten the comprehensiveness of publicly funded healthcare.

On to our second in-practice consideration. The provision of healthcare covered by VHI may very well draw financial and personnel resources from publicly funded healthcare systems and consequently lead to the displacement of care. This is already an issue with out-of-pocket payments for healthcare costs (Fenton, 2011). In case of VHI, it will be even more pressing, given that it brings expensive treatments into the reach of many more citizens. This is especially problematic in countries with egalitarian healthcare systems that have no private sector to deliver the type of expensive care covered by VHI, such as Sweden and the Netherlands.

This means that expensive treatments must be administered in public hospitals, and the same holds for all the monitoring and treatment of side effects, adverse events and complications, and for the provision of follow-up care that is associated with these treatments. While VHI might cover the treatment itself, the publicly funded healthcare system might have to shoulder the ancillary costs associated with the treatment. In theory, this problem can be overcome by having VHI cover ancillary costs as well. In practice, however, charging ancillary costs is difficult, because it involves an extra administrative burden (Jackson, 2010: 411–412). Also, these costs are often difficult to accurately determine. For instance, it may be difficult to distinguish care provided for the treatment of (long-term) complications of VHI-funded treatments from care that would otherwise also have been provided.

Still the biggest problem may be the limited total number of hospital beds and the difficulty to attract sufficient healthcare staff that many countries are currently facing. If the volume of VHI-funded care becomes significant, this may lead to longer waiting lists for everyone and to the displacement of publicly funded care. Finally, the public funding of medical education is yet another way in which VHI would be subsidized by public means (Sagan and Thomson, 2016: 27). These are all substantial reasons against the introduction of VHI.

Our third in-practice consideration turns on the fact that differential treatment of patients who are equally in need but differ in VHI status may run too much against the egalitarian ethos of doctors. Strong expressions of such ethos can be found, for example, in Bloor, who argues that out-of-pocket payments go against the founding principle of the NHS that states that access to treatments ‘shall not depend on whether [patients] can pay for them or on any other factor irrelevant to real need’ (Bloor, 2008). A recent interview study shows that Dutch physicians are generally decidedly against allowing patients to use private funds, expressing views such as ‘I believe that everyone should have the same opportunities, independent from how much money or connections they have’ (Bomhof *et al.*, 2022). Importantly, having to act against their egalitarian ethos may eventually be harmful to physicians’ moral integrity.

In this respect, it is relevant to acknowledge that even if insurers were to offer VHI, that does not necessarily mean that public hospitals and their physicians would be obliged to deliver VHI-funded care. Especially when it comes to the provision of healthcare over and above the basic package, insurers and hospitals, depending on the legal landscape of their country, may be free to negotiate their contracts. The wish to continue to work in line with their egalitarian ethos may be a valid reason for physicians working in public hospitals to be unwilling to deliver care covered by VHI.

The same holds for all the other practical implications discussed in this section: they may constitute reasons for hospitals not to deliver VHI-funded healthcare. Whether or not hospitals will attach decisive weight to these reasons will also depend on their weighing of the entirety of considerations, both in principle and in practice, that we have discussed so far. We will give our tentative weighing in the next, concluding section.

## Concluding Discussion

Publicly funded healthcare systems basically have three options for dealing with treatments that are effective, yet too expensive for inclusion in the basic package of healthcare. First, they can avoid hard choices and silently stretch their priority-setting criteria, because not including an effective treatment will not be accepted by citizens. However, stretching these criteria necessarily leads to either displacement of other, cost-effective healthcare, or cuts in the funding for other important social goods, such as education and housing. Second, they can strictly adhere to their priority-setting criteria and disallow or disincentivize VHI. In that way, they will safeguard relational equality, at the cost of foregone medical benefits to patients and the curtailing of citizens’ liberty to spend their after-tax money in ways they see fit. Third and finally, they can be strict as well, but allow VHI, and thereby safeguard the medical benefits and liberty for those who can afford VHI, at the cost of increased relational inequality between citizens.

Clearly, each of these alternatives has its own major disadvantages and therefore, none is an attractive option. Option one provides most opportunity to make the disadvantages invisible, so as to avoid societal backlash. As a result, when governments are unwilling to face tough choices, they are easily set on this path. But this option is not in line with governmental obligations to provide equal opportunities for its citizens. So let us assume a government that is strict. In that case, is it morally desirable that insurers offer VHI for expensive non-reimbursed treatments?

The answer depends on the considerations discussed in our paper. First, we found that the telic egalitarian objection to unequal access to treatment due to different ability to pay for VHI does not succeed. Second, whether there may nevertheless be a justice-based objection against VHI depends on the actual degree of relational inequality that would result from its presence. Although citizens’ experiences of loss of self-respect or their perceptions of society as insufficiently caring (loss of fraternity) would have to be determined empirically, it seems safe to assume that there will be some degree of relational inequality in a publicly funded healthcare system with VHI for expensive treatments.

Given that assumption, it is important that the introduction of VHI should not undermine publicly funded healthcare in any way. Otherwise, those who are unable to afford VHI would not only face relational inequality, but also see their healthcare deteriorate. Accordingly, if we allow VHI, we should carefully monitor how the healthcare system develops, prevent ‘crowding out’ or the draining of budget and personnel from publicly funded healthcare, and ensure that citizen commitment to the public system does not deteriorate. The basic package should remain comprehensive, high quality *and* the same for all citizens. VHI should cover only *additional*, insufficiently cost-effective services. If it is not possible to meet these requirements, VHI should be disallowed, or at least strongly disincentivized. In such cases, we submit, it is clear that the medical benefits and liberty of the well-off who can afford VHI do not outweigh the decline of publicly funded healthcare and the emergence of relational inequality resulting from VHI.

In the introduction, we made the assumption of a sufficiently just society, in which income differences are by and large justifiable. It is now time to relax this assumption and see how this alters our weighing of values. While theories of justice do not give clear guidance, some relational egalitarians, for example, argue that rather egalitarian income distributions follow from the notion that citizens participate as equals in reciprocal co-operations by which they jointly produce all the goods a society needs (Schemmel, 2011; Scanlon, 2018). In any case, *if* income differences are unjust, then the differential outcomes resulting from different ability to pay for VHI mean that existing injustices are maintained or even exacerbated. It would be clearly unfair if one’s low income were undeserved and if, in addition, this undeserved inability to pay were the cause that one cannot receive medical treatment. The translation of unjust income distributions into differential access to medical treatments would then serve as a decisive reason against VHI. Therefore, under such circumstances, governments should not allow VHI unless they can find ways to make sure that any citizen who prefers taking out VHI has the means to do so.

In conclusion, we have identified several plausible pathways through which a practice of differential access to VHI could create injustice, and, moreover, exacerbate existing injustice. This should make governments cautious in allowing VHI. However, some degree of relational inequality could potentially be accepted in the form of lessened self-respect and weakened bonds of fraternity between citizens, because evaluative errors are involved in their emergence and hence they are not fully reasonable. Moreover, proper weight should be given to citizens’ liberty to spend their money so as to seek additional medical care. Therefore, perhaps, a combination of strict priority setting with allowing VHI for non-reimbursed insufficiently cost-effective treatments could be the least bad alternative in dealing with the increasing costs of medical treatments.^13^

## Footnotes

1
https://www.rijksoverheid.nl/actueel/nieuws/2023/03/28/geneesmiddel-trodelvy-niet-in-basispakket [accessed 12 May 2023].

2 Listed price in the EU: 327.000 EUR, see https://www.apmhealtheurope.com/freestory/0/64434/major-german-payers-sign-pay-for-performance-agreements-on-car-ts [accessed 12 May 2023].

3 For an example of such insurance, see https://www.aviva.co.uk/help-and-support/managing-your-policy/health/cancer-essentials/ [accessed 12 May 2023].

4 In several countries, citizens can already take out voluntary health insurance for dental services, physiotherapy and the like. However, here we focus on very expensive treatments.

5 That does not mean that many treatments are currently inaccessible to patients in the countries this paper focuses on. For example, the UK has a Cancer Drug Fund for treatments that are not yet approved by its healthcare institute NICE. See https://www.england.nhs.uk/cancer/cdf/ [accessed 12 May 2023].

6 For the insight that we should also compare with healthy citizens, see also (Färdow *et al.*, 2019: 7) who, however, do not discuss it in connection to the leveling-down objection.

7 We do not think that Rawl’s idea of non-comparing groups as a buffer against a loss of self-respect will be of much help here (Rawls, 1971: 388, 470). By way of (social) media-coverage, painful instances of the consequences of differential access to VHI will be highly visible to all members of society.

8
https://www.zorginstituutnederland.nl/publicaties/verslag/2021/04/23/verslag-adviescommissie-pakket-acp-23-april-2021 [accessed 12 May 2023].

9
https://zoek.officielebekendmakingen.nl/stcrt-2020-23796.html [accessed 12 May 2023].

10 ‘Baby Liam heeft twee miljoen euro nodig voor het duurste medicijn ter wereld’. Noordhollands Dagblad, 18 February 2021. https://www.noordhollandsdagblad.nl/cnt/dmf20210218_59447263 [accessed 12 May 2023], and NOS, NOS, 30 January 2020, ‘Ouders baby Jayme hopen op levensreddend medicijn: “Het is dit of niets”’. https://nos.nl/artikel/2321000-ouders-baby-jayme-hopen-op-levensreddend-medicijn-het-is-dit-of-niets [accessed 12 May 2023].

11 One indication is the fact that in its information provision about cancer treatments, the UK charity Macmillian Cancer Support explicitly refers to the option of private payment or additional voluntary health insurance to cover non-reimbursed cancer care. See https://www.macmillan.org.uk/cancer-information-and-support/treatment/your-treatment-options/what-you-can-do-if-a-treatment-is-not-available [accessed 12 May 2023]. Furthermore, we have not been able to find references to a societal debate in the UK on these issues. We have tracked the references to papers written on the, back then around 2008, debated issue of out-of-pocket payments in the NHS (e.g. Bloor, 2008; Desai *et al.*, 2009) and the debate seems to have faded away rather quickly.

12
https://www.england.nhs.uk/publication/individual-funding-requests-for-specialised-services-a-guide-for-patients/ [accessed 4 July 2023].

13 We thank the audience of our presentation of an earlier version of the paper at the 2022 ISPH Priorities conference in Bergen for their valuable feedback. We also thank Wynand van de Ven and Piet Calcoen for valuable discussions on the practicalities, ethics and politics of voluntary health insurance. We thank Charlotte Bomhof, Eline van der Meer, and Maartje Schermer for helpful feedback on an earlier version. Finally, we thank two reviewers for this journal for their very helpful and constructive comments.
